# Gene signature based on B cell predicts clinical outcome of radiotherapy and immunotherapy for patients with lung adenocarcinoma

**DOI:** 10.1002/cam4.3561

**Published:** 2020-10-24

**Authors:** Linzhi Han, Hongjie Shi, Yuan Luo, Wenjie Sun, Shuying Li, Nannan Zhang, Xueping Jiang, Yan Gong, Conghua Xie

**Affiliations:** ^1^ Department of Radiation and Medical Oncology Zhongnan Hospital of Wuhan University Wuhan Hubei China; ^2^ Department of Thoracic and Cardiovascular Surgery Zhongnan Hospital of Wuhan University Wuhan Hubei China; ^3^ Department of Biological Repositories Zhongnan Hospital of Wuhan University Wuhan Hubei China; ^4^ Human Genetics Resource Preservation Center of Hubei Province Human Genetics Resource Preservation Center of Wuhan University Zhongnan Hospital of Wuhan University Wuhan Hubei China; ^5^ Hubei Key Laboratory of Tumor Biological Behaviors Hubei Cancer Clinical Study Center Zhongnan Hospital of Wuhan University Wuhan Hubei China

**Keywords:** immunotherapy, lung adenocarcinoma, prognosis, radiotherapy, tumor‐infiltrating B lymphocytes

## Abstract

Lung adenocarcinoma (LUAD) is the most common and lethal cancer worldwide. Radiotherapy (RT) is widely used at all stages of LUAD, and the development of immunotherapy substantially enhances the survival of LUAD patients. Although the emerging treatments for LUAD have improved prognosis, only a small fraction of patients can benefit from clinical therapies. Thereby, approaches assessing responses to RT and immunotherapy in LUAD patients are essential. After integrating the analysis of RT, immunization, mRNA, and clinical information, we constructed a signature based on 308 tumor‐infiltrating B lymphocyte‐specific genes (TILBSig) using a machine learning method. TILBSig was composed of 6 B cell‐specific genes (PARP15, BIRC3, RUBCNL, SP110, TLE1, and FADS3), which were highly associated with the overall survival as independent factors. TILBSig was able to differentiate better survival compared with worse survival among different patients, and served as an independent factor for clinical characteristics. The low‐risk TILBSig group was correlated with more immune cell infiltration (especially B lineages) and lower cancer stem cell characteristics than the high‐risk group. The patients with lower risk scores were more likely to respond to RT and immunotherapy. TILBSig served as an excellent predicator for prognosis and response to immunotherapy and RT in LUAD patients.

## Graphical abstract

Our studies identified a 6‐genes signature TILBSig based on tumor‐infiltrating B lymphocyte specific genes, which might serve as predictors of survival and response to immunotherapy and radiotherapy for LUAD patients.

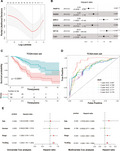

## 1 INTRODUCTION

Lung cancer is the most lethal malignancy, with a mortality rate ranking the highest (18.4% of the total number of deaths).^1^ The histologic subtype of approximately 85% lung cancer is non‐small cell lung cancer (NSCLC), in which lung adenocarcinoma (LUAD) accounts for up 40% cases.^2^, ^3^, ^4^ Among multiple clinical treatments including surgical resection, chemotherapy, and targeted therapy,^5^, ^6^ radiotherapy (RT) is used at all stages of LUAD as a routine treatment. Approximately 77% lung cancer patients received RT,^7^ which elicits DNA damage and immunogenic cell death, induces tumor neoantigen release, and activates immune system.^8^ However, the therapeutic effects of RT is unsatisfactory due to radioresistance. A considerable proportion of LUAD patients has relapses followed by RT.^9^ Therefore, seeking for appropriate therapies for patients of radioresistance is significant.

Tumor microenvironment (TME) includes tumor infiltrated immune cells, among which, tumor‐infiltrating lymphocytes (TILs) have profound effects on clinical outcomes.^10^ It was reported that B cells had both positively and negatively regulatory effect on cancer progression.^11^ B cells exerted antitumor function by increasing T cell immunity,^12^ enhancing interferon‐γ production, and assisting antitumor effects of nature killer (NK) cells.^11^, ^13^ In addition, B cells also inhibited immune responses, supported tumor growth, and promoted angiogenesis in TME.^11^, ^14^ Moreover, B cells were reported to be correlated with extended prognosis in LUAD patients.^15^, ^16^, ^17^ However, the roles of B cells are little known in LUAD patients with RT.

In our study, we first divided the patients into the RT sensitivity (RS) and RT resistance (RR) groups using consensus clustering method, and found that tumor‐infiltrating B lymphocytes (TIL‐Bs) had significantly difference between groups. TIL‐B‐specific genes were then identified based on B cell lines and other 19 immune cell lines. Furthermore, we established a 6‐genes signature based on TIL‐B‐specific genes (TILBSig). The tumor‐infiltrating immune cells, immune‐associated molecules, and cancer stem cell (CSC) characteristics were compared between the high‐ and low‐risk patients. The signature was validated and served as a predictive factor for the response of LUAD patients to immune checkpoint inhibitors (ICIs) treatment and RT.

## 2 MATERIALS AND METHODS

### 2.1 Data acquisition and preprocessing

The work flow chart is displayed in Figure S1. The gene expression data and the corresponding clinical information of LUAD patients were collected from the Gene Expression Omnibus (GEO; http://www.ncbi.nlm.nih.gov/geo) and The Cancer Genome Atlas (TCGA; https://portal.gdc.cancer.gov). After removing the patients with follow‐up time less than 30 days or no clear information about RT, 423 patients and their mRNA sequencing data (FPKM normalized) were selected from TCGA database. Among of the 57 TCGA‐LUAD patients receiving RT, 46 patients had records of radiation responses. Two external validation sets (GSE37745 and GSE30219) representing independent studies of LUAD were obtained from GEO based on Affymetrix HG‐U133_Plus 2.0 platform. A total of 188 LUAD patients (GSE37745: 105 LUAD patients; GSE30219: 83 LUAD patients) with follow‐up time longer than 30 days were included after examining the corresponding survival information of either data set. The gene expression profiles of LUAD cell lines were downloaded from the GEO database under accession number GSE57083 profiled by the Affymetrix HG‐U133_Plus 2.0 platform. The raw CEL files of GEO LUAD patients and LUAD cell lines were collected and uniformly processed using the Robust Multichip Average (RMA) algorithm for background correction and normalization. One genomic and transcriptomic data set (GSE78220) of patients with metastatic melanoma treated with ICIs was downloaded from GEO and analyzed to determine the predictive value of our risk model (Table S1). Finally, we normalized all data sets using the min‐max method.

### 2.2 Differential expression analysis of mRNAs between B cell lines and other immune cell lines

The raw CEL files of B cells and 19 other immune cell types were screened out from the GEO database‐based Affymetrix HG‐U133_Plus 2.0 platform under the accession numbers GSE6863, GSE8059, GSE13906, GSE23371, GSE25320, GSE27291, GSE27838, GSE28490, GSE28698, GSE28726, GSE37750, GSE39889, GSE42058, GSE49910, GSE51540, GSE59237, and GSE63626. All of the raw data were then background‐corrected and quantile‐normalized using RMA algorithm. R package “limma” was performed to identify differential expression genes (DEGs) between B cell lines and other immune cell lines based on |LogFC| >1 and FDR <0.05.

### 2.3 Radiosensitivity clustering

According to Kim et al. grouping method,^18^ we classified 423 TCGA‐LUAD patients into the RR and RS groups using consensus clustering (*k* = 2) with a subsampling ratio of 0.8 and a total of 1000 permutation tests. Chi‐square tests were used to compare clinical features between the RR and RS groups.

### 2.4 Construction of TILBSig

A total of 423 TCGA‐LUAD patients were randomly divided into the training set (n = 212) and the internal testing set (n = 211). Chi‐square test showed that the baseline data of train and test groups were balanced (Table S2). Univariate and least absolute shrinkage and selection operator (LASSO) COX regression analysis were used to select the independent risk mRNAs that were highly expressed in B cell lines and downregulated in other immune cell lines. By multivariate Cox regression analysis, we constructed the TILBSig. With survival and survminer R package, survival analysis of TILBSig was illustrated. The survivalROC R package was performed to construct time‐dependent receiver operating characteristic (ROC) curve. GSE37745 and GSE30219 were regarded as two independent validation sets to verify the feasibility of the signature.

### 2.5 Gene set enrichment analysis (GSEA)

To understand the underlying function of TILBSig, GSEA was performed for functional enrichment analysis of Gene Ontology (GO) and Kyoto Encyclopedia of Genes and Genomes (KEGG). *p* < 0.05 was considered significant.

### 2.6 Statistical analysis

The Kaplan–Meier method was used to generate survival curves for the subgroups in each data set. The Wilcoxon rank‐sum test was applied for comparisons of two groups, and Chi‐square test was used for comparisons of more than two groups. Univariate Cox analysis was performed to calculate the hazard ratios for univariate analyses. Independent prognostic factors were determined using a multivariate Cox regression model. Correlations were computed using the Spearman method, and their significance was assessed using a correlation test.

## 3 RESULTS

### 3.1 Identification of radiosensitive patients and B cell‐specific genes

**FIGURE 1:**
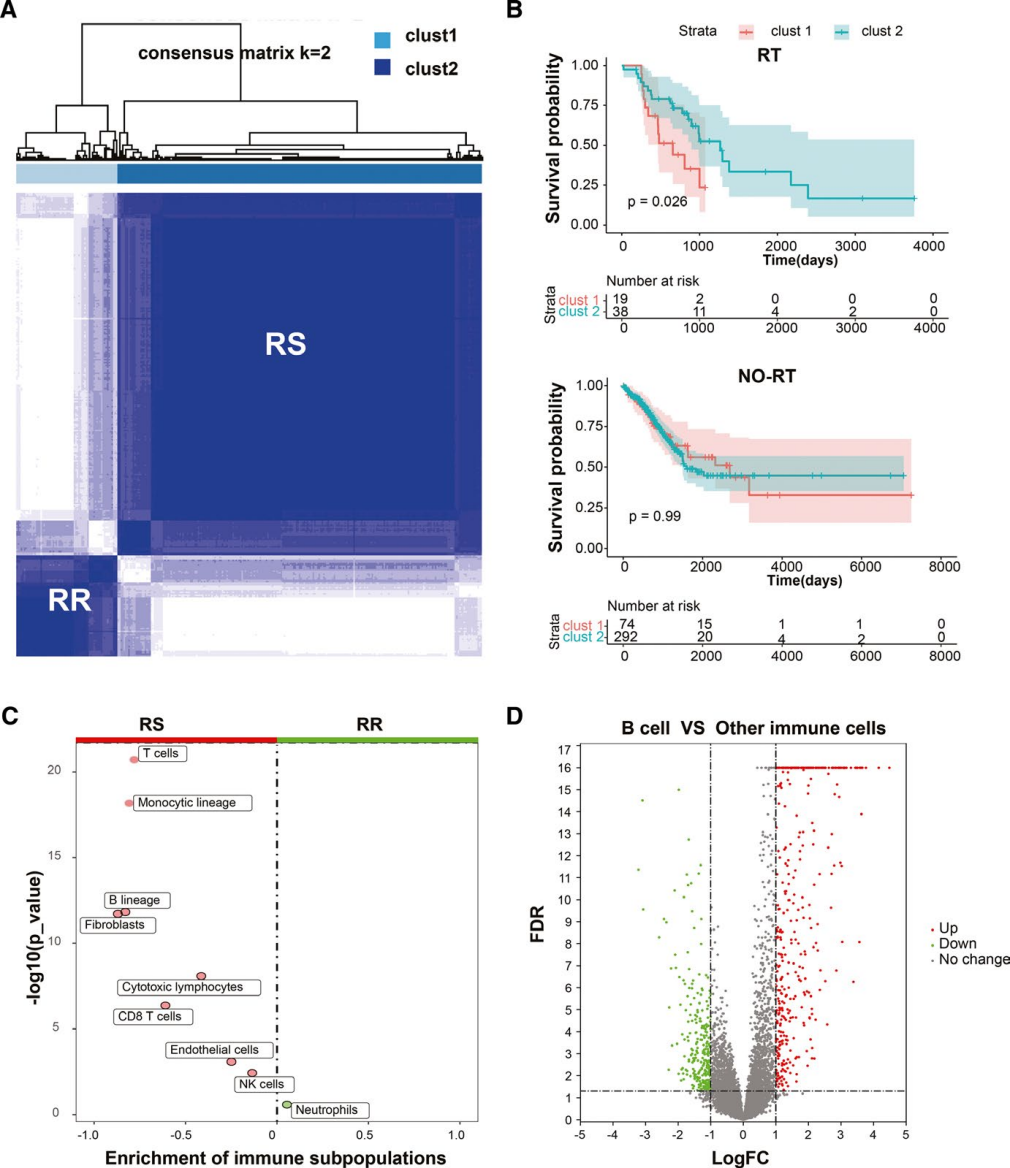
Definition process of the RS versus RR groups and the identification of TIL‐B‐specific genes. A, Consensus clustering for LUAD patients. B, Kaplan–Meier curves for the OS in receipt of RT. C, Difference of immune subpopulations fraction between the RR and RS groups. D, Volcano plot for the differential genes between B cell and other immune cells.

Consensus cluster analysis was used to stratify the patients. After that, the 423 TCGA‐LUAD patients were divided into the clust1 and clust2 groups. Compared with patients who received RT in clust1, patients receiving RT in clust2 had better prognosis. Therefore, clust2 with good prognosis was defined as RS group, while clust1 was defined as RR group. (Figure 1A, B). The clinical characteristics including gender, pathological stage, T stage, and RT of LUAD patients were significantly different between the RR and RS groups (Chi‐square test; gender, *p* = 0.0005; pathological stage, *p* = 0.042; T stage, *p* = 0.034; RT, *p* = 0.04, Table 1). MCP counter algorithm allows the robust quantification of the absolute abundance of eight immune and two stromal cell populations. Its R package was used to estimate the abundance of immune cell infiltration in the 423 samples.^19^ Difference of infiltrated immune cells between the RS and RR groups is demonstrated in Figure 1C. T cells, monocytic lineages, B lineages, fibroblasts, cytotoxic lymphocytes, CD8T cells, endothelial cells, and NK cells were enriched in the RS group, while neutrophils was enriched in the RR group. Moreover, R package “limma” was performed to identify DEGs between B cell lines and 19 other immune cell lines (all of the immune cell line data sets were obtained from GEO). A total of 393 genes upregulated in B cells and downregulated in other immune cells were selected as the B cell‐specific genes based on |LogFC| >1 and FDR <0.05. (Figure 1D).

**TABLE 1:** Patient characteristics.

Variable	RR (n = 93)	RS (n = 330)	Total (n = 423)	X‐squared	*p*‐value
Gender				12.082	**0.0005**
Female	36	197	233		
Male	57	133	190		
Age				0.597	0.74
<65 y	44	149	193		
≥65 y	46	174	220		
Unknown	3	7	10		
Stage				8.9879	**0.042**
I	40	195	235		
II	27	75	102		
III	16	42	58		
IV	7	14	21		
Unknown	3	4	7		
T				9.2337	**0.034**
T1	22	127	149		
T2	53	166	219		
T3	10	22	32		
T4	4	9	13		
Unknown	4	6	10		
N				1.905	0.75
N0	57	217	274		
N1	18	60	78		
N2	14	36	50		
N3	0	2	2		
Unknown	4	15	19		
M				3.5747	0.16
M0	62	206	268		
M1	7	13	20		
Unknown	24	111	135		
RT				4.2105	**0.04**
Yes	19	38	57		
No	74	292	366		

The bold *p*‐values indicate the statistically significant difference.

### 3.2 Identification and validation of B cell‐specific gene signature

**FIGURE 2:**
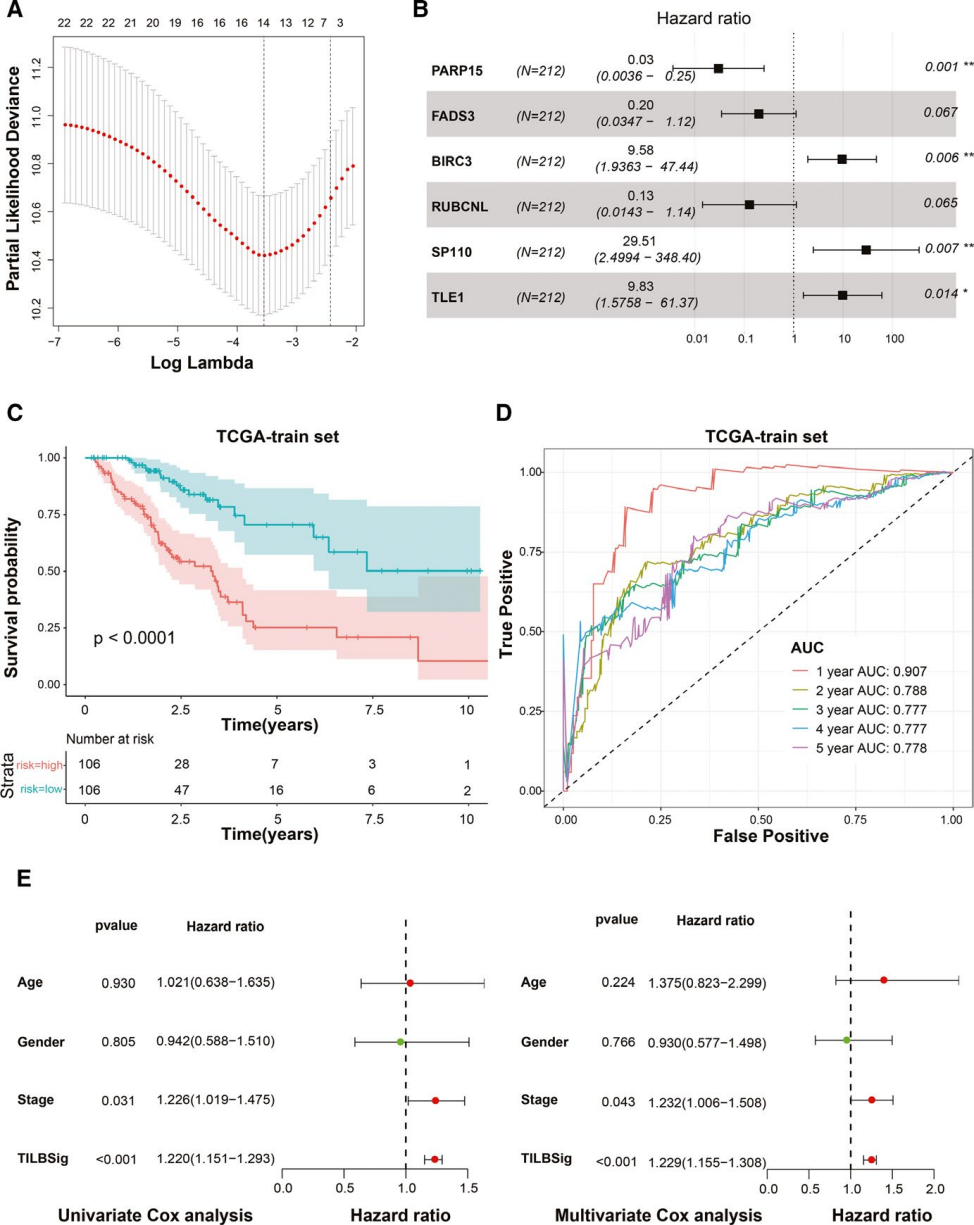
Development of the TILBSig in the TCGA training set. A, LASSO Cox regression analysis for B cell‐specific genes. B, Forest plot visualizing the HRs of multivariate Cox analysis of six mRNAs in the TILBSig with the OS. C, Survival analysis between the high‐ and low‐risk groups stratified by the TILBSig in TCGA training set. D, Time‐dependent ROC curve for TCGA training set. E, Forest plot visualizing the HRs of univariate Cox and multivariate Cox analysis of the TILBSig and clinicopathological factors in TCGA training set

To elucidate the potential implications of B cell‐specific genes in prognosis, RT, and immunotherapy, 308 genes of the 393 upregulated genes in B cells from GEO database were found in TCGA data set. Univariate analysis was used to identify 22 prognosis‐related genes, and LASSO COX regression analysis was then used to select 14 independent risk genes. At last, six genes were screened out to construct a B cell‐specific gene signature using multivariate Cox regression analysis. The signature was composed of 6 B cell‐specific genes (PARP15, FADS3, RUBCNL, BIRC3, SP110, and TLE1), all of which were independent risk factors for the overall survival (OS) (Figure 2A,B). The risk score of the signature for OS was identified: risk score = (−3.503) × (expression level of PARP15) + (−1.624) × (expression level of FADS3) + (−2.0592) × (expression level of RUBCNL) + 2.26 × (expression level of BIRC3) + 3.385 × (expression level of SP110) + 2.286 × (expression level of TLE1). According to the medial risk score, patients in the TCGA training set were divided into the high‐ and low‐risk groups. The low‐risk group had better survival than the high‐risk group (*p* < 0.0001, Figure 2C). The area under ROC curve (AUC) for 1‐, 2‐, 3‐, 4‐, and 5‐year OS were 0.907, 0.788, 0.777, 0.777, and 0.778 (Figure 2D). Stage and TILBSig were independent predicators of the OS in univariate cox analysis (*p* = 0.031, *p* < 0.001) and multivariate cox analysis (*p* = 0.043, *p* < 0.001, Figure 2E).

**FIGURE 3:**
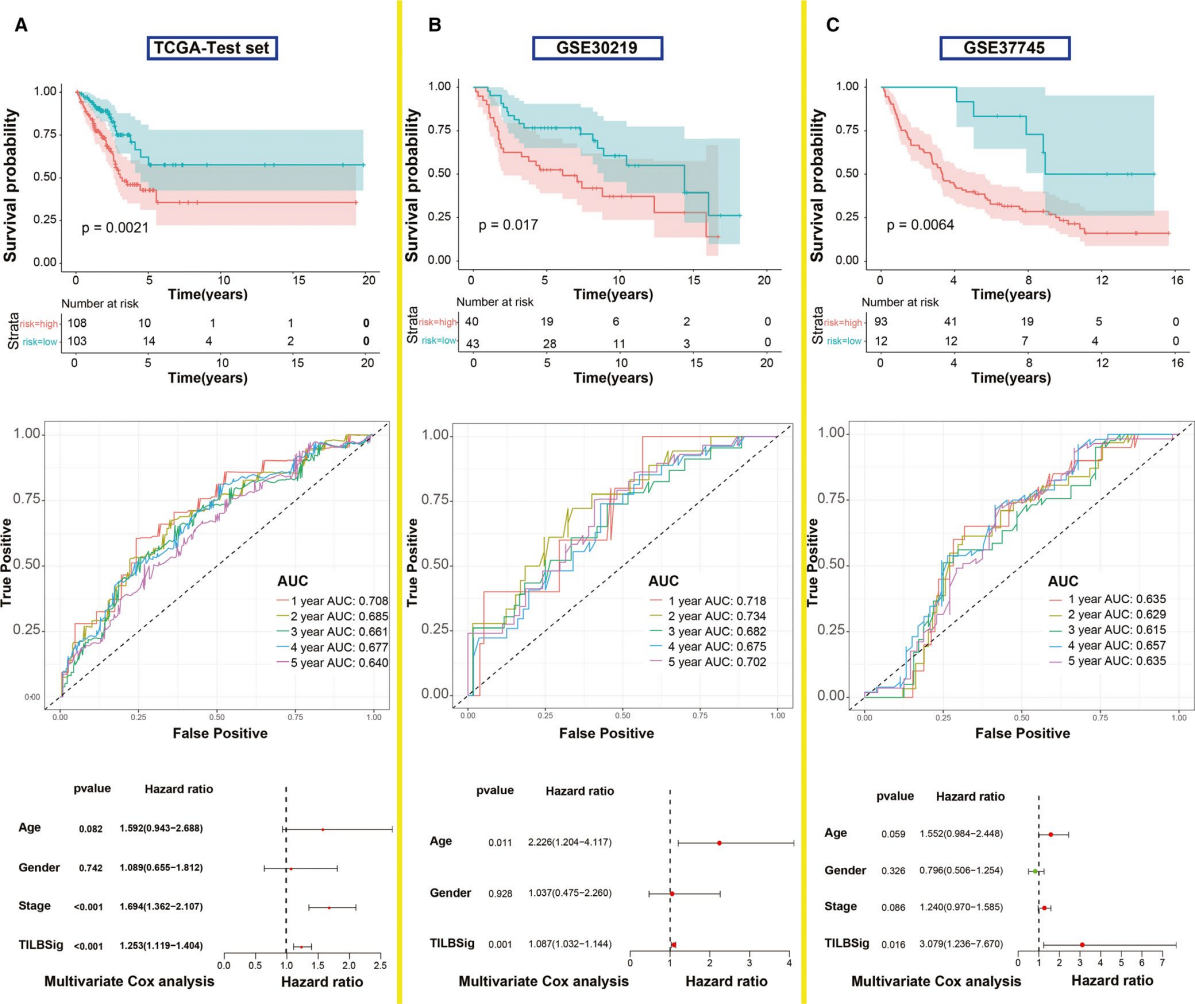
Validation of the TILBSig in the TCGA testing set and two external independent data sets. A, Kaplan–Meier survival curve, time‐dependent ROC curve, and multivariate Cox analysis of the TILBSig and clinicopathological factors in TCGA testing set. B, Kaplan–Meier survival curve, time‐dependent ROC curve, and multivariate Cox analysis of the TILBSig and clinicopathological factors in GSE30219. C, Kaplan–Meier survival curve, time‐dependent ROC curve, and multivariate Cox analysis of the TILBSig and clinicopathological factors in GSE37745 cohort

The survival predictive ability of TILBSig was validated in TCGA testing set, GSE30219 and GSE37745. The prognosis of the low‐risk group was better than that of the high‐risk group. The area under ROC curve (AUC) for 1‐, 2‐, 3‐, 4‐, and 5‐year OS were all over 0.6 for three validation gene sets. Stage and TILBSig were independent predicators of the OS in univariate cox analysis and multivariate cox analysis (Figure 3A–C, Figure S2). Therefore, TILBSig could serve as an excellent biomarker for OS prediction.

### 3.3 Relation between TILBSig and tumor microenvironment

**FIGURE 4:**
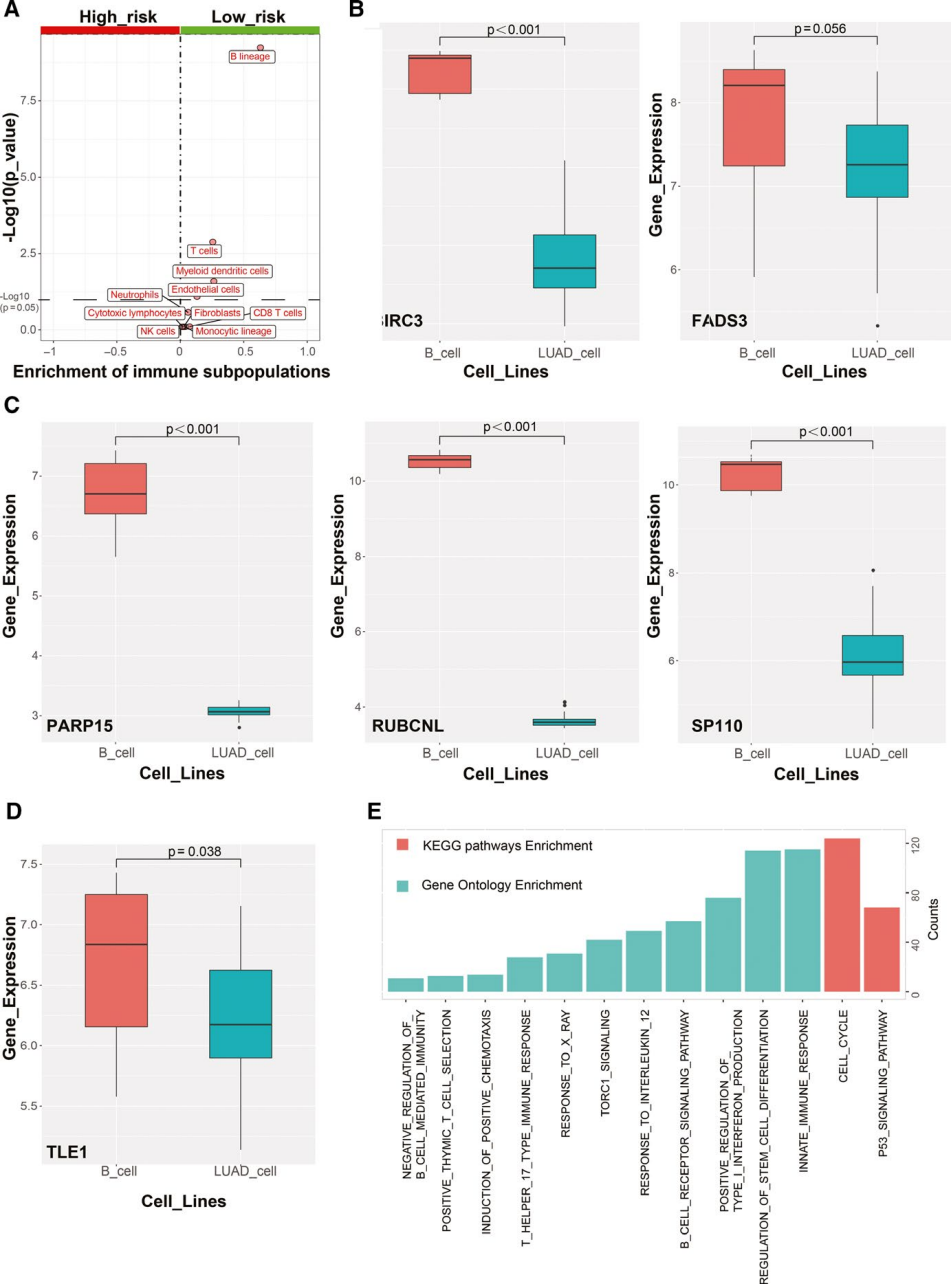
Functional analysis of TILBSig. A, Volcano plots for difference of immune subpopulations fraction between the high‐ and low‐risk groups. B–D, Boxplots for the differential of genes expression between B cell lines and LUAD cell lines. E, Enrichment analysis of GO and KEGG pathways for TILBSig‐related genes.

**FIGURE 5:**
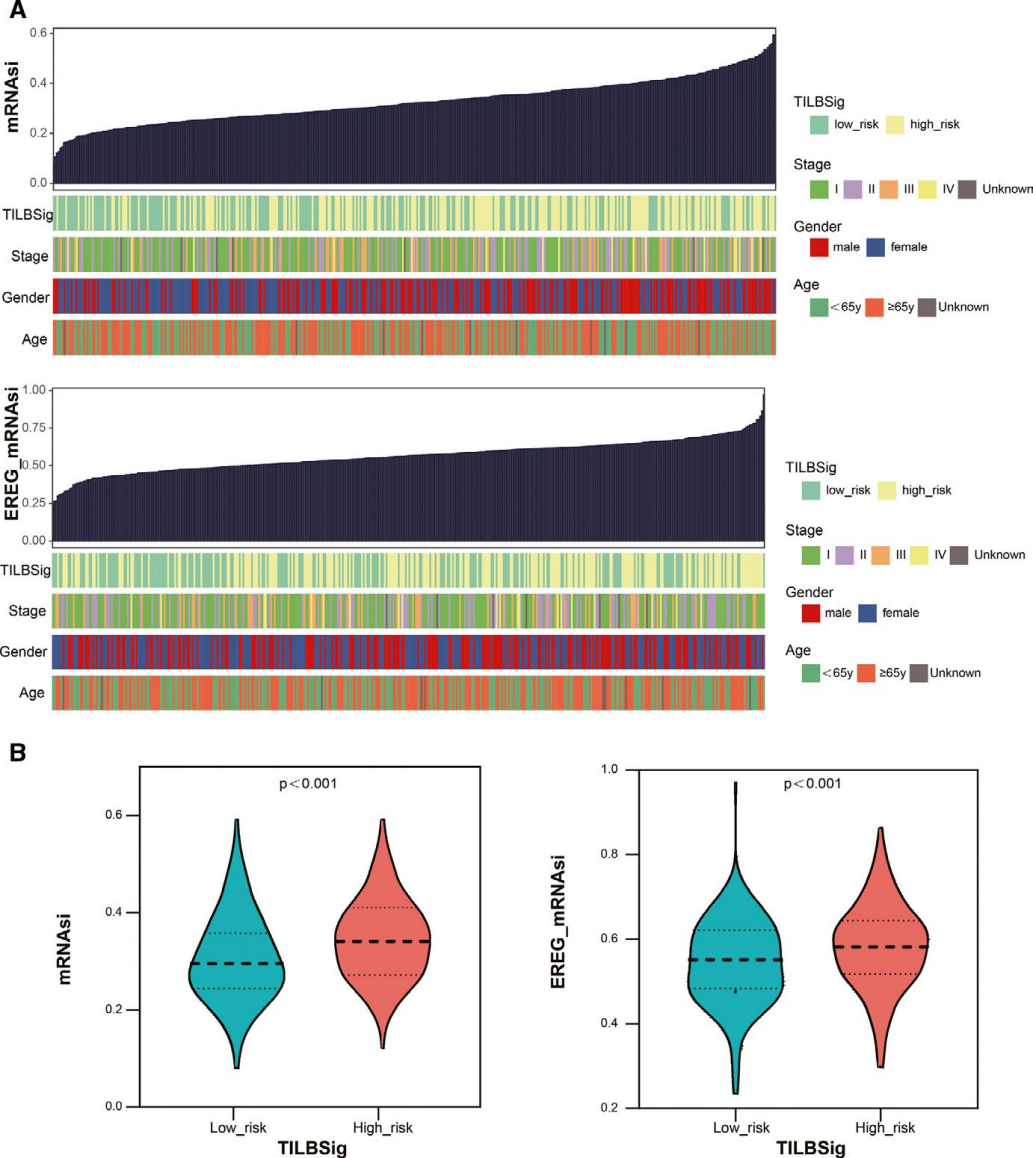
Tumor stemness index characterization of the TILBSig. A, An overview of the association between known clinical features and TILBSig and mRNAsi/EREG‐mRNAsi. B, Violin plots of mRNAsi/EREG‐mRNAsi distribution in the high‐ and low‐risk groups

In order to find association between TILBSig and immune infiltration, 10 immune subpopulations were analyzed using MCP counter. B lineages, T cells, myeloid dendritic cells, endothelial cells, fibroblasts, CD8+ T cells, monocytic lineages, neutrophils, cytotoxic lymphocytes, and NK cells were enriched in the low‐risk group. In contrast, no immune subpopulations were enriched in the high‐risk group (Figure 4A). Furthermore, the expression of these six genes were particularly upregulated in B cell lines rather than LUAD cell lines, suggesting that the six genes were specifically expressed in B cells (Figure 4B–D). GSEA showed that TILBSig was closely associated with B cell receptor signaling pathway, regulation of stem cell differentiation, response to X ray, and innate immune response (Figure 4E). Considering the fact that CSCs were positively correlated with tumorigenesis and radioresistance,^20^ samples in TILBSig were ranked based on mRNAsi (tumor stemness index based on mRNA expression) and EREG‐mRNAsi (tumor stemness index based on stem cell epigenetic regulation‐related genes) (Figure 5A). The low‐risk group had lower CSC characteristics than the high‐risk group (Figure 5B). As a result, TILBSig was capable of recognizing patients with high CSC characteristics. This was consistent with our enrichment analysis results.

### 3.4 TILBSig as a predicator to radiotherapeutic and immunotherapeutic responses

**FIGURE 6:**
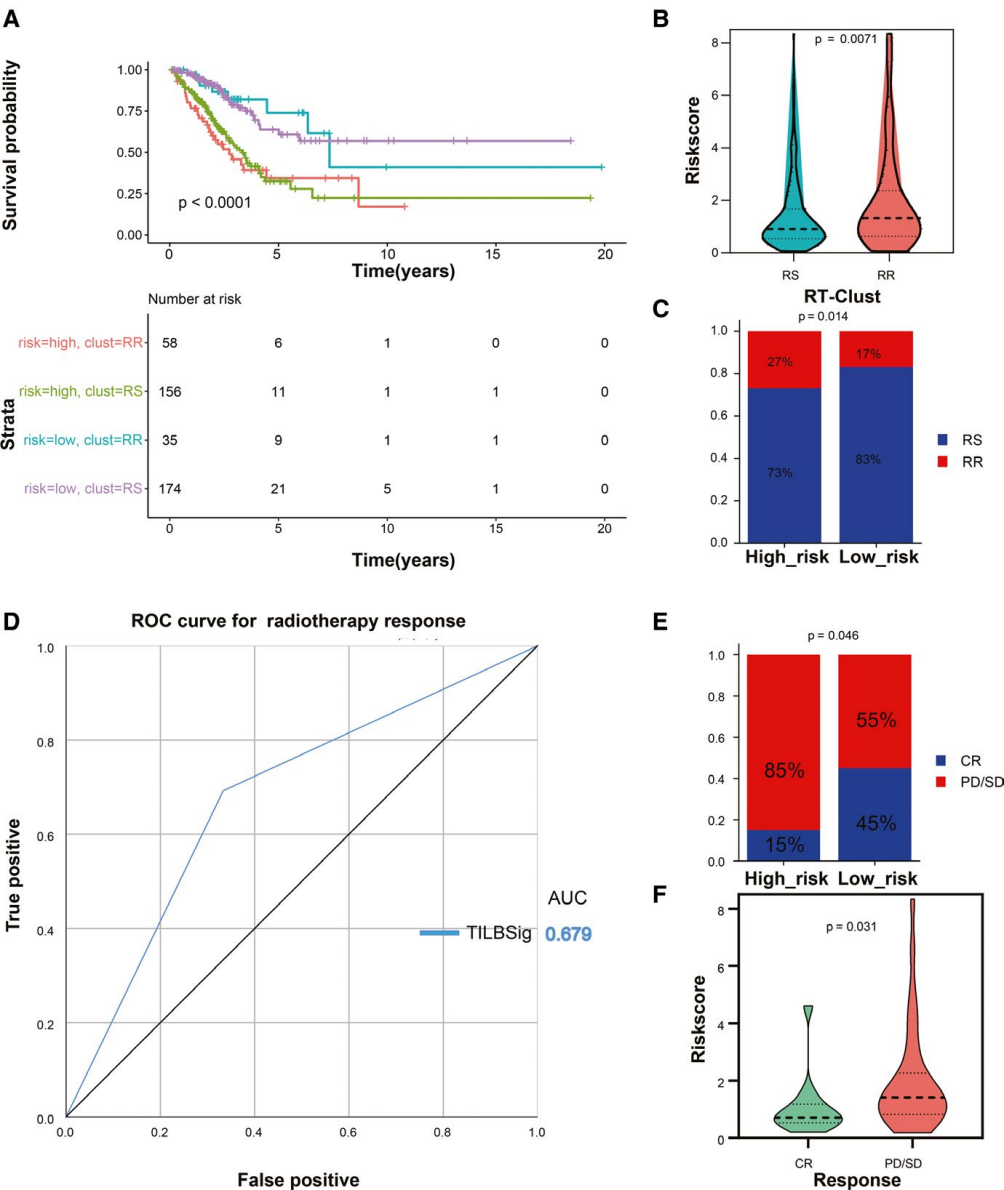
TILBSig could predict RT response in LUAD patients. A, Survival analysis among four patient groups stratified by the TILBSig and RT‐Clust (RR and RS groups). B, Violin plot of TILBSig risk score distribution in the RR and RS groups (Wilcoxon rank‐sum test, *p* = 0.0071). C, Rate of the RR and RS patients in the high‐ or low‐risk groups in the TCGA‐LUAD patients (Chi‐square test, *p* = 0.014). D, ROC curve measuring the predictive value of the TILBSig to RT response. (E) Rate of clinical response to RT in the high‐ and low‐risk groups in the TCGA‐LUAD patients treated with RT (complete response [CR], stable disease [SD], progressive disease [PD]; Chi‐square test, *p* = 0.046). F, Violin plot of TILBSig risk score distribution in different clinical response to RT (Wilcoxon rank‐sum test, *p* = 0.031).

The correlation between TILBSig, clinical response of RT, and immunotherapy were subsequently explored. Patients from TCGA‐LUAD data set were stratified to the high‐risk RR, high‐risk RS, low‐risk RR, and low‐ risk RS groups based on TILBSig. The low‐risk RS group had the best prognosis compared with the other three groups, whereas the high‐risk RR group showed the worst survival (Figure 6A). Consistently, the RR groups had higher risk scores than the RS groups (Figure 6B), and patients with lower risk scores showed higher radiosensitive rate (83%) than those with higher risk scores (73%, *p* = 0.014, Figure 6C). Afterward, ROC curve revealed that TILBSig obtained an AUC of 0.679 for predicting response to RT (Figure 6D). More importantly, patients in the low‐risk groups had higher complete response (CR) rate (85%) for RT than those in the high‐risk groups (55%, *p* = 0.046, Figure 6E). Comparison results also indicated that patients with CR had much lower risk scores than those with stable disease (SD) or progressive disease (PD, *p* = 0.031, Figure 6F).

**FIGURE 7:**
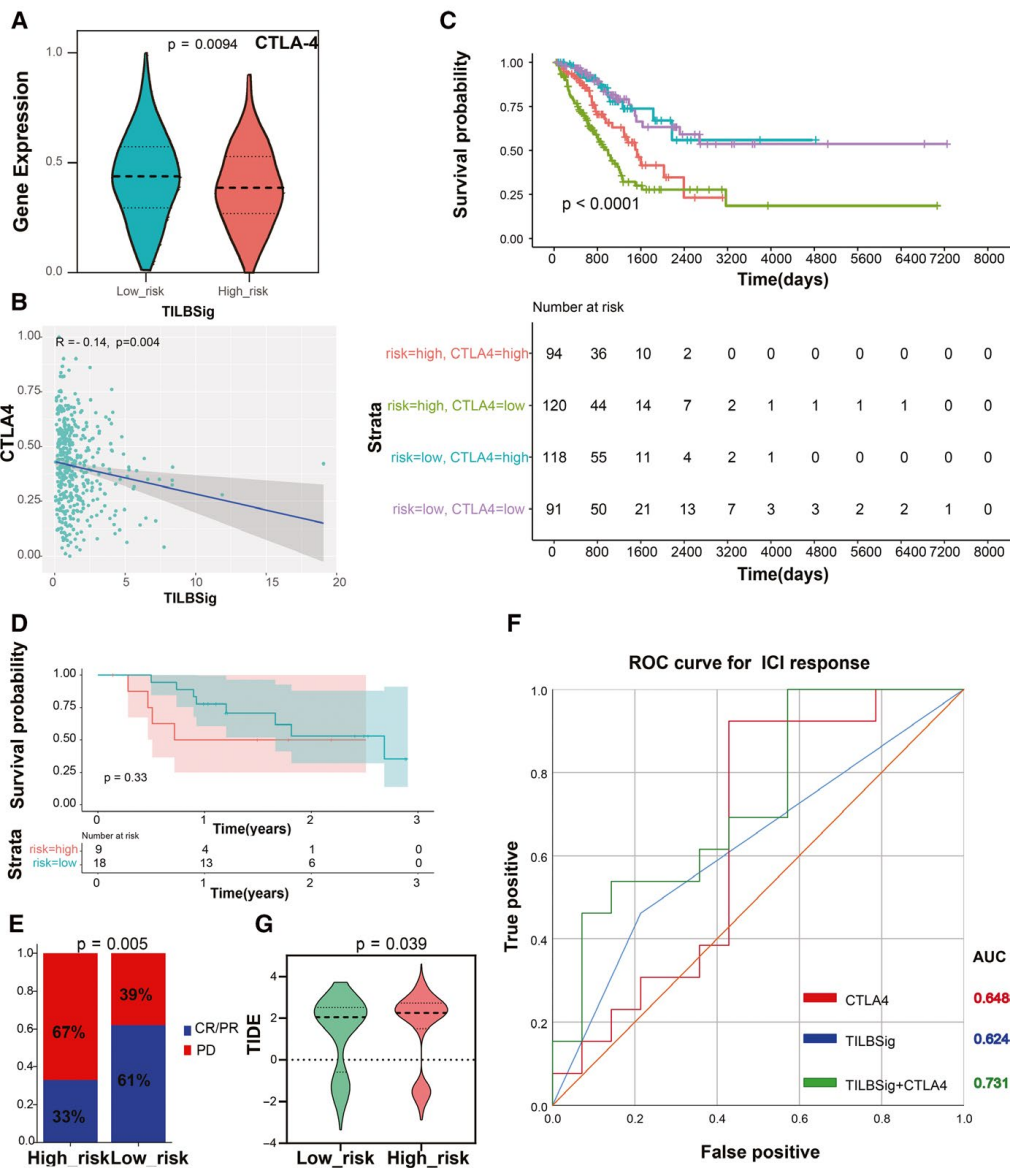
TILBSig could predict the immunotherapeutic benefits. A, Violin plot of CTLA‐4 expression distribution in the low‐ and high‐risk groups (Wilcoxon rank‐sum test, *p* = 0.0094). B, Association between CTLA‐4 expression levels and TILBSig risk scores. C, Survival analysis among four patient groups stratified by the TILBSig and CTLA‐4 expression. According to the median expression of CTLA‐4, the patients were divided into high‐ and low‐CTLA‐4 groups. D, Kaplan–Meier curves for patients with high and low risk in the GSE78220 cohort. E, Rate of clinical response to immunotherapeutic in the high‐ and low‐risk groups in the GSE78220 cohort (complete response [CR], partial response [PR], progressive disease [PD]; Chi‐square test, *p* = 0.005). F, ROC curves measuring the predictive value of the TILBSig, CTLA‐4 expression, and combination of TILBSig and CTLA‐4 expression in the GSE78220 cohort. G, Differences in TIDE scores between the high‐ and low‐risk groups (Wilcoxon rank‐sum test, *p* = 0.039).

Because of the significant immune infiltration difference between the high‐ and low‐risk groups, we investigated the predictive ability of TILBSig for immunotherapeutic responses. As the expression levels of programed cell death‐1 (PD1) and programed death‐ligand 1 (PD‐L1) showed no significant difference between the high‐ and low‐risk groups, another important immune checkpoint molecule, cytotoxic T lymphocyte‐associated antigen 4 (CTLA‐4) was included to obtain the cross talk between TILBSig and immunotherapy. The expression of CTLA‐4 was negatively related with risk scores (*p* < 0.05, Figure 7A,B). Patients with lower risk scores and higher CTLA‐4 had the longer survival time, while the patients with higher risk scores and lower CTLA‐4 had the worse prognosis (*p* < 0.0001, Figure 7C). Current studies on tumor‐associated antigens were most successful in melanoma, which was a tumor with stronger immunogenicity than others.^21^ If immunotherapy was not effective in melanoma, it is likely to be ineffective in other tumors such as rectal cancer, lung cancer, stomach cancer, and so on. Considering the lack of immunotherapy data for lung cancer, we selected the melanoma immunotherapy data set (GSE78220) to verify TILBSig. Survival analysis suggested that the patients with lower risk scores in metastatic melanoma treated with ICIs had favorable clinical outcome (Figure 7D). Furthermore, the response rate of immunotherapy was higher in the low‐risk (61%) group (33%, *p* = 0.005, Figure 7E). Finally, AUC for CTLA‐4 inclined to 0.731 after combing with TILBSig, which was 0.648 for CTLA‐4 and 0.624 for TILBSig in ROC curve for ICI response prediction (Figure 7F). Tumor Immune Dysfunction and Exclusion (TIDE) was a computational method to model two primary mechanisms of tumor immune evasion, which could predict ICB responses (http://tide.dfci.harvard.edu/). The lower the TIDE score, the better the effect of immunotherapy.^22^, ^23^ Our result showed that the TIDE score of the low‐risk group was significantly lower than that of the high‐risk group, indicating that the low‐risk group could benefit more from immunotherapy. In conclusion, TILBSig might be a potential factor for distinguishing LUAD patients who respond to RT and immunotherapy.

## 4 DISCUSSION

TME has important roles in modulating tumor progression, in which TILs play paramount functions. Cytotoxic T lymphocytes and NK cells have enormous effects on immunotherapy. Recently, TIL‐Bs were reckoned as vital factors in antitumor immunity,^24^, ^25^ including generating antibodies and anticancer cytokines, presenting cancer‐related antigens and killing tumor cells directly in TME.^26^, ^27^ Additionally, patients with lung or ovarian cancer showed favorable prognosis when they had high ratios of B cells, mature dendritic cells, and CD8+ T cells instead of only CD8+ T cells.^26^, ^28^, ^29^ Thus, TIL‐Bs in tumors might serve as potential markers for survival prediction. Moreover, there were increased evidences suggesting that RT had a key role in treatment of most LUAD patients.^7^ Radiation exerts lethal DNA damage in irradiated tumor cells, which improves outcomes of these patients in terms of local control.^8^ However, not all patients could benefit from RT, with a considerable of LUAD patients showing radioresistance and suffering from tumor metastasis.^9^ Combined application of tumor RT and ICIs has achieved enormous progress in enhancing antitumor treatment outcome.^30^, ^31^, ^32^, ^33^ After introducing ICIs to clinical treatment, combination of ICIs and RT in LUAD increased the chances of distant cancer regression in tumor metastatic region.^34^ This phenomenon derived from immunostimulatory effects of RT, which stimulated release of tumor‐associated antigens and immune molecules.^35^ Due to the inextricable association between these two therapies, we creatively constructed TILBSig aiming at identifying patients who respond to both RT and ICIs.

In our study, patients from TCGA‐LUAD were divided into the RS and RR groups using consensus clustering method. Clinical characteristics analysis showed that gender and stage were significantly associated with the RR and RS groups, which indicated that female patients and patients at lower stages might be more sensitive to RT (Table 1). Furthermore, we found that TIL‐Bs were significantly different between the RR and RS groups, suggesting that TIL‐Bs served as an important factor in LUAD RT. A 6‐B cell‐specific‐genes signature based on TIL‐B‐specific genes was constructed. In the TILBSig, 3 B cell‐specific genes (PARP15, FADS3, and RUBCNL) were correlated with better prognosis, while the other three (BIRC3, SP110, and TLE1) were correlated with poor survival. PARP15, polymerase family member 15, was originally confirmed as a risk‐related gene in diffuse large B cell lymphomas, and might be potential predictors of hematological toxicity associated with RT for acute myeloid leukemia, cervical cancer, nasopharynx cancer, and tongue cancer.^36^, ^37^, ^38^, ^39^, ^40^ FADS3, as a fatty acid desaturase, was located along with FADS1 and FADS2,^41^ which were reported to be associated with the occurrence and development of NSCLC and colon cancer.^42^, ^43^ Promoter methylation of RUBCNL was identified as a potential biomarker for early diagnosis of cervical cancer.^44^ BIRC3, baculoviral IAP repeat‐containing protein 3, as an apoptosis inhibitor, was overexpressed in multiple cancers and led to development of malignant tumors.^45^, ^46^, ^47^, ^48^, ^49^, ^50^ Upregulation of BIRC3 promoted prostate cancer development and inhibited NK cell activities.^51^, ^52^ SP110 was reported as a commonly deregulated gene in mammary cancer and an early inducement in melanoma and nonmelanoma skin cancer.^53^, ^54^ TLE1 promoted EMT in A549 lung cancer cells via suppressing E‐cadherin.^55^, ^56^ All the six genes were potentially prognostic markers in LUAD.

To investigate the OS prediction of TILBSig, ROC curve of TILBSig was estimated. Results for AUC demonstrated that TILBSig had a good association with clinical prognosis in both training and validation sets. Further univariate and multivariate cox analysis confirmed that TILBSig could act as an independent factor for the OS prediction. By comparing enrichment of immune subpopulations between the high‐ and low‐risk groups, B cells were the most abundant cell in the low‐risk group. Other gene signature in LUAD also showed B cells were highly infiltrated in the low‐risk group. A 10‐immune‐related‐genes signature constructed by Jiaona Zhu et al. ^57^ and IPSLUAD signature developed by Jie He ^58^ were predicted the prognosis of patients well. Both of the two gene signatures suggested that B cells were highly infiltrated in the low‐risk group, but the role of B cells in tumor immune infiltration was not explored. Compared with their signatures, TILBSig had a higher AUC value in terms of predicting prognosis, indicating a better predictive ability of TILBSig. In addition, the six TILBSig genes were highly expressed in B cell lines compared with LUAD cell lines. These results suggested that the TILBSig might be a potent biomarker of both B cell infiltration and patient prognosis.

RT was reported to stimulate immune cell recruitment to radiation field and to improve the antitumor effects of the immune system.^59^ RT could trigger damaged double‐stranded DNA via eliciting immunogenic cell death.^60^ Through Cyclic GMP‐AMP synthase (cGAS)‐stimulator of interferon genes (STING) pathway, RT promoted production of type 1 interferon bridging innate immune response and adaptive immune response.^61^ Moreover, Jones and Shuxian et al. found that radiation‐induced tumor regression via stimulating immunity activation and increasing B and NK cell infiltration.^62^, ^63^ All of these results were consistent with our research. In our study, GO and KEGG analysis revealed that TILBSig was highly associated with positive thymic T cell selection, response to X ray, positive regulation of type 1 interferon production, and regulation of stem cell differentiation. We speculated that the better prognosis of patients in the low‐risk group might be related to the activation of the immune system by RT. In addition, several studies illustrated that CSCs had pivotal roles in tumor progression and radioresistance. Better local control was observed in tumors with lower levels of CSCs when applied to the same radiation dose.^64^, ^65^ In accordance with impacting radiosensitivity ability of CSCs, higher CSC index in the high‐risk group validated our TILBSig as a marker to differentiate RR and RS patients (Figure 5). Finally, by comparing the difference of RT response between the high‐ and low‐risk groups, we found that the low‐risk TILBSig group possessed higher RS and CR rates than the high‐risk group, which partially validated our previous conclusion (Figure 6).

ICIs were popularly used in cancer patients to improve prognosis, especially PD‐1, PD‐L1, and CTLA‐4 antibodies.^66^, ^67^ Despite the satisfactory outcome, only a limited proportion of patients benefited from ICIs. As a result, assessing patients with potential clinical responses to ICIs is urgent. Previous researches indicated that the immunomodulatory interaction between B cell infiltration and checkpoint gene expression might affect the prognosis of patients and be related to the response of patients to immunotherapy.^24^ Consistently, Beth and Amaria et al. showed that B cell subsets in melanoma and renal cell carcinoma were highly infiltrated in patients who responded to ICIs compared with nonresponse patients, implying that TIL‐Bs might act as a proxy to predict ICI therapeutic responses.^68^, ^69^ Considering the important roles of TIL‐Bs in patients’ responses to ICIs, we further explored the relationship among TIL‐Bs, immune checkpoints, and ICIs. After examining the gene expression difference of immune checkpoints between the high‐ and low‐risk groups, CTLA‐4 had significant difference. Furthermore, our results indicated that TILBSig could distinguish responders from nonresponders well for ICI‐treated patients in metastatic melanoma immunotherapy data set. Especially, when we combined CTLA‐4 expression with TILBSig to predict patients’ response to ICIs, the predictive ability (AUC = 0.731) was significantly higher than that of CTLA4 expression (AUC = 0.648) or TILBSig (0.624) alone. These results suggested the TILBSig might represent tumor immunosuppression status and predict ICI responses in lung cancer patients. Therefore, TILBSig not only had prognostic values for ICI‐treated patients, but also had the ability to distinguish responders from nonresponders. According to our TILBSig, patients in the low‐risk group would benefit more from RT combined with immunotherapy. Altogether, our research showed that TILBSig had a good ability to predict the responses to immunotherapy and RT.

## 5 CONCLUSIONS

In summary, our study constructed an immune/radiation‐relevant gene signature based on 6 tumor‐infiltrating B lymphocytes‐specific genes, and the gene signature had a good ability to predict prognosis, RT, and immunotherapeutic responses in LUAD patients. The patients in the low‐risk group might be more likely to benefit from the combined therapy of RT and immune checkpoint inhibitors.

## CONFLICTS OF INTEREST

All authors declare no conflicts of interest.

## AUTHOR CONTRIBUTIONS

LH, YG, and CX designed the study. LH, HS, YL, and WS collected the data and clinical information. LH, SL, NZ, and XJ contributed to the statistical analysis. LH drafted the manuscript. YG and CX revised the article. All authors read and approved the final version.

## Supporting information

Fig S1‐S2

Table S1‐S2

## Data Availability

All data in this study are available upon request from the corresponding author.
