# Supplementary material for: Gene signature based on B cell predicts clinical outcome of radiotherapy and immunotherapy for patients with lung adenocarcinoma
Source: Cancer Med. 2020 Oct 24;9(24):9581–94. doi: 10.1002/cam4.3561 (PMC7774727; doi:10.1002/cam4.3561)
Supplement: Supplementary file 1 — Fig S1‐S2 [file CAM4-9-9581-s001.pdf]

# Support Information (Figure)

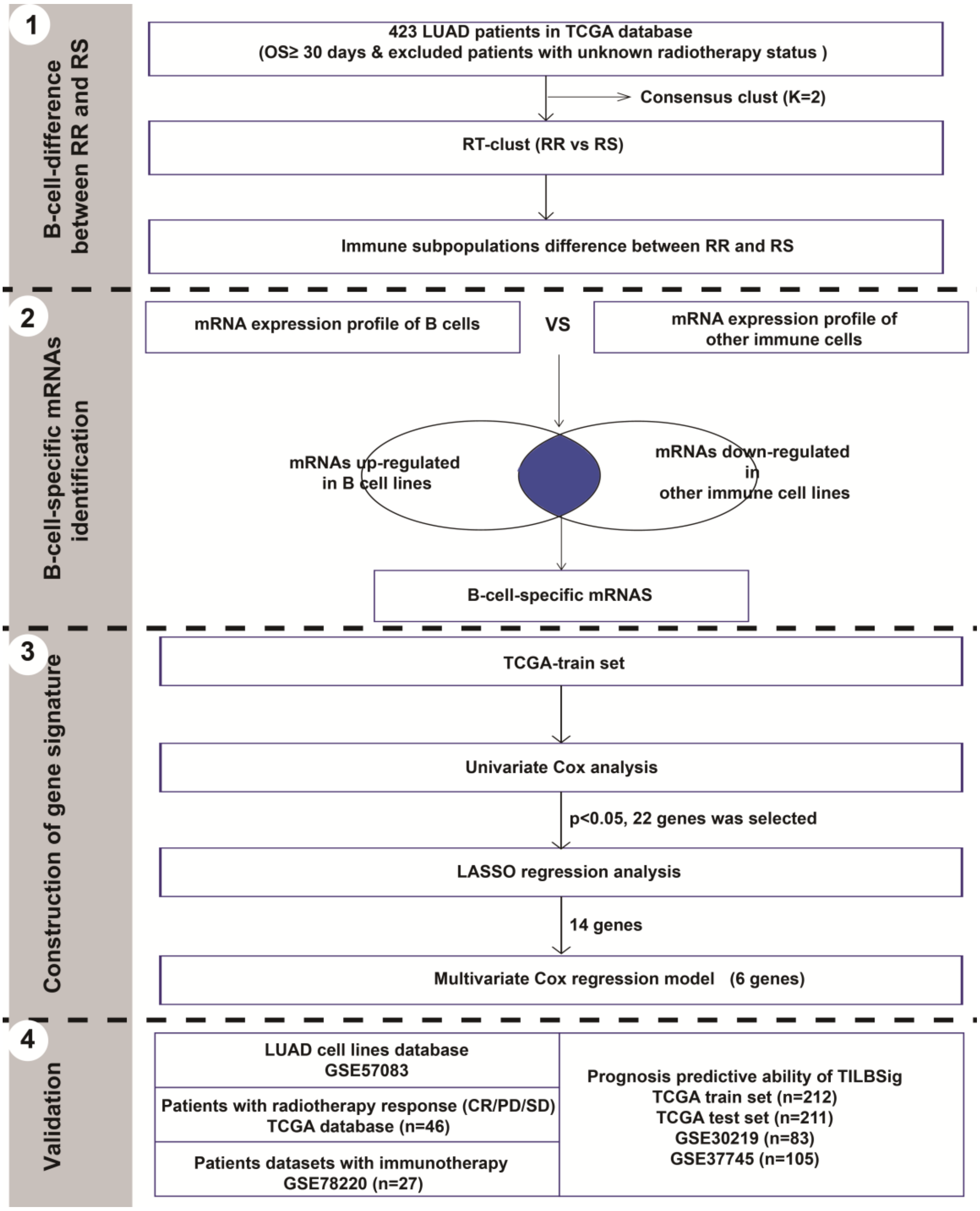


**FIGURE S1** The flow chart of the study.

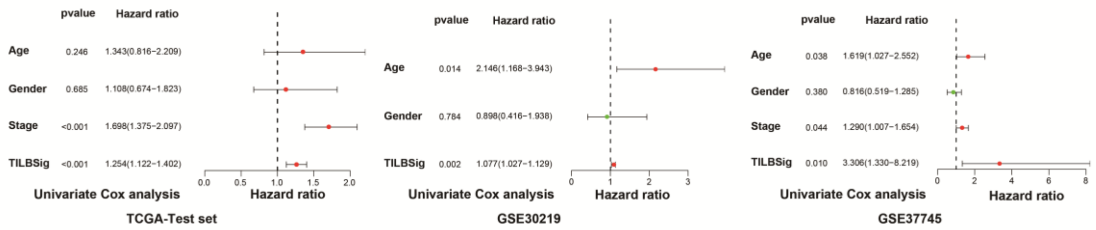


**FIGURE S2** Univariate Cox analysis of the TILBSig and clinicopathological - factors in TCGA testing set, GSE30219 and GSE37745 cohort.
