# Supplementary material for: Gene signature based on B cell predicts clinical outcome of radiotherapy and immunotherapy for patients with lung adenocarcinoma
Source: Cancer Med. 2020 Oct 24;9(24):9581–94. doi: 10.1002/cam4.3561 (PMC7774727; doi:10.1002/cam4.3561)
Supplement: Supplementary file 2 — Table S1‐S2 [file CAM4-9-9581-s002.docx]

Support Information (Table)

| **Data sets** | **Source** | **PMID** | **Sample size for**  **each group** | **Platform/Technology** |
| --- | --- | --- | --- | --- |
| **Discovery set (n = 423)** | TCGA | \ |  | lllumina HiSep |
| **External validation set**  **(n = 188)** | GSE37745 | 23032747 | 105 | Affymetrix  Human Genome  U133 Plus 2.0 Array |
|  | GSE30219 | 23698379 | 83 | Affymetrix  Human Genome  U133 Plus 2.0 Array |
| **Immunetherapy response**  **validation set (n = 27)** | GSE78220 | 26997480 | 27 | Illumina HiSeq 2000 |
| **LUAD cell lines** | GSE57083 | \ | 43 | Affymetrix  Human Genome  U133 Plus 2.0 Array |
| **Immune cell lines** | GSE6863, GSE8059, GSE13906,  GSE23371, GSE25320, GSE27291,  GSE27838, GSE28490, GSE28698,  GSE28726, GSE37750, GSE39889,  GSE42058, GSE49910, GSE51540,  GSE59237, GSE63626 | 32382761 | \ | Affymetrix  Human Genome  U133 Plus 2.0 Array |

Table S1 Data sets obtained from TCGA and GEO.

| **Variable** | **Train set (n = 212)** | **Test set (n = 211)** | **Total (n = 423)** | **X-squared** | **p-value** |
| --- | --- | --- | --- | --- | --- |
| **Vital status** |  |  |  | 0.4879 | 0.48 |
| **Alive** | 141 | 148 | 289 |  |  |
| **Dead** | 71 | 63 | 134 |  |  |
| **Gender** |  |  |  | 0.2837 | 0.59 |
| **Female** | 120 | 113 | 233 |  |  |
| **Male** | 92 | 98 | 190 |  |  |
| **Age** |  |  |  | 0.3977 | 0.53 |
| **<65y** | 98 | 105 | 203 |  |  |
| **≥65y** | 114 | 106 | 220 |  |  |
| **Stage** |  |  |  | 0.6125 | 0.96 |
| **I** | 117 | 118 | 235 |  |  |
| **II** | 50 | 52 | 102 |  |  |
| **III** | 29 | 29 | 58 |  |  |
| **IV** | 12 | 9 | 21 |  |  |
| **Unknown** | 4 | 3 | 7 |  |  |
| **Radiotherapy** |  |  |  | 0.7631 | 0.3824 |
| **Yes** | 25 | 32 | 57 |  |  |
| **No** | 187 | 179 | 366 |  |  |

Table S2 Patient characteristics between train and test group.
